# EXPERIENCE OF LGBT PATIENTS AND FAMILY WITH HOSPICE AND PALLIATIVE CARE

**DOI:** 10.1093/geroni/igz038.2323

**Published:** 2019-11-08

**Authors:** Gary L Stein, Cathy Berkman

**Affiliations:** 1 Wurzweiler School of Social Work-Yeshiva University, New York, New York, United States; 2 Graduate School of Social Service-Fordham University, New York, New York, United States

## Abstract

This study examines the degree to which hospice and palliative care staff observe or perceive inadequate, disrespectful, or abusive care to LGBT patients and family members. A cross-sectional study using an online survey completed by 865 providers, including social workers, physicians, nurses, and chaplains. Among respondents, 55% reported that LGB patients were more likely to experience discrimination at their institution than non-LGB patients; 24% observed discriminatory care; 65% reported that transgender patients were more likely than non-transgender patients to experience discrimination; 20% observed discrimination to transgender patients; 14% observed the spouse/partner of LGBT patients having their treatment decisions disregarded or minimized; and 13% observed the spouse/partner being treated disrespectfully. Findings reported also include: institutional non-discrimination policy, staff training, intake procedures, and comfort in assessing LGBT status. Implications for future research, policy, and practice will be presented.

